# High-Frequency Analysis of the Cerebral Physiological Impact of Ketamine in Acute Traumatic Neural Injury

**DOI:** 10.1089/neur.2024.0146

**Published:** 2025-02-20

**Authors:** Davis McClarty, Logan Froese, Tobias Bergmann, Kevin Y. Stein, Amanjyot S. Sainbhi, Abrar Islam, Nuray Vakitbilir, Noah Silvaggio, Izabella Marquez, Alwyn Gomez, Frederick A. Zeiler

**Affiliations:** ^1^ Undergraduate Medicine, College of Medicine, Rady Faculty of Health Sciences, University of Manitoba, Winnipeg, Manitoba, Canada.; ^2^ Department of Clinical Neuroscience, Karolinska Institutet, Stockholm, Sweden.; ^3^ Department of Biomedical Engineering, Price Faculty of Engineering, University of Manitoba, Winnipeg, Manitoba, Canada.; ^4^ Department of Human Anatomy and Cell Science, Rady Faculty of Health Sciences, University of Manitoba, Winnipeg, Manitoba, Canada.; ^5^ Undergraduate Biosystems Engineering, Price Faculty of Engineering, University of Manitoba, Winnipeg, Manitoba, Canada.; ^6^ Section of Neurosurgery, Department of Surgery, Rady Faculty of Health Sciences, University of Manitoba, Winnipeg, Manitoba, Canada.; ^7^ Pan Am Clinic Foundation, Winnipeg,Manitoba, Canada.

**Keywords:** acute brain injuries, ketamine, sedation, traumatic brain injury

## Abstract

Acute traumatic neural injury, also known as traumatic brain injury (TBI), is a leading cause of death. TBI treatment focuses on the use of sedatives, vasopressors, and invasive intracranial pressure (ICP) monitoring to mitigate ICP elevations and maintain cerebral perfusion pressure (CPP). While common sedatives such as propofol and fentanyl have significant side effects, ketamine is an attractive alternative due to its rapid onset and cardiovascular stability. Despite these benefits, ketamine’s use remains controversial due to historical concerns about increasing ICP. Using high-frequency monitoring, this retrospective study compared cerebral pressure-flow dynamics in patients with moderate/severe TBI who received ketamine with those who did not. Statistical analysis included descriptive statistics, comparisons within and between patients receiving ketamine, and evaluation of physiological response around incremental dose changes in ketamine. Various cerebral physiological indices were analyzed, including ICP, CPP, regional cerebral oxygen delivery, intracranial compliance, and cardiovascular reactivity metrics. A total of 122 patients were studied, with 17 receiving ketamine (median age: 37 years) and 105 not receiving ketamine (median age: 42 years). Results indicated higher median ICP in the ketamine group compared with the no ketamine group (9.05 mmHg and 14.00 mmHg, respectively, *p* = 0.00017); however, this is likely due to differences in patient characteristics and injury severity between the groups. No significant differences were observed in any other index of cerebral pressure-flow dynamics or between any incremental dose change condition. These findings suggest that ketamine does not significantly impact cerebral pressure-flow dynamics, challenging historical concerns about its use in patients with TBI.

## Introduction

Acute traumatic neural injury (also known as traumatic brain injury [TBI]) is one of the leading causes of death, impacting 50 million people a year worldwide.^1^ TBI is composed of two components: the initial injury occurring at the time of the incident, known as the primary injury, and the subsequent damage due to cascading physiological factors, known as the secondary injury. Secondary injury manifests as progressive edema, metabolic derangements, and inflammation, all of which disrupt cerebral blood flow regulation and impair oxygen and nutrient delivery.^2^ Prevention of primary injury focuses on primary prevention measures (helmets, seatbelts, etc.) as little can be done to repair the primary injury. However, secondary injuries are theoretically amenable to treatment, with edema reduction, neuronal protection, and mediation of impaired cerebral blood flow control (termed cerebral autoregulation [CA] or cerebrovascular reactivity [CVR]) being the focus of current guideline-based therapies.^3–6^

The acute treatment of patients with TBI focuses on mitigating invasive intracranial pressure (ICP) elevations while maintaining cerebral perfusion pressure ([CPP], CPP = ICP − arterial blood pressure [ABP]).^3^ To achieve these treatment goals, therapeutics such as vasopressors (norepinephrine, phenylephrine) and high-dose intravenous sedative agents (propofol, fentanyl, and midazolam) are administered. Sedative agents achieve their effects through suppression of neuronal activity, reducing metabolic demands with the goal of neuronal tissue preservation. However, commonly utilized sedative agents carry significant side-effect profiles in high doses used for TBI care, including systemic hypotension, bradycardia, and mitochondrial dysfunction. As such, alternative agents with different mechanisms of action have been desired in the critical care management of the patients with TBI.

Ketamine’s rapid onset of action, dissociative anesthesia properties, and pain modulation make it a useful agent for procedural sedation and induction.^7^ Moreover, ketamine does not cause vasodilation or have a negative chronotropic impact on the heart.^8^ These characteristics make it an attractive anesthetic when trying to maintain perfusion pressure. Despite these advantages, the use of ketamine in TBI has not been widely adopted in the field of neurocritical care.^9^ The current neuroanesthesia literature associates the use of ketamine with uncontrollable increases in ICP.^10–13^ Consequently, physicians often avoid ketamine when there is a concern for elevated ICP, a particular concern in TBI populations. However, there is minimal evidence in the literature that supports the notion that ketamine increases ICP. Rather, there is a small body of evidence that demonstrates ketamine does not increase ICP and may be associated with a decrease in ICP.^14–20^ This small body of evidence is based on low-frequency physiological data (hourly or daily mean data), without time-linked waveform physiology and pharmacologic information. Low-frequency data collection reduces temporal resolution, limiting accurate capture of relationships between physiological variables and clinical interventions. These conflicting viewpoints and lack of high frequency data highlight that the physiological impact of ketamine on cerebrovascular physiology is poorly understood. Therefore, there exists a need for the use of high-frequency monitoring to better understand the impact of ketamine on ICP and other measures of cerebrovascular physiology/CVR.

Advances in biomedical signal processing have led to the ability to monitor ICP, CPP, regional cerebral oxygen saturation (rSO_2_), and CVR indices over time, in a high-frequency format.^21,22^ Combining pharmacologic treatment information with high-frequency cerebral physiological data facilitates bridging the knowledge gaps regarding the impact of ketamine on cerebral physiological pressure-flow dynamics. Leveraging the existing archived data from the moderate/severe TBI database at the University of Manitoba (UM), the aim of this project is to investigate the impact of ketamine on cerebral pressure-flow dynamics in patients with moderate/severe TBI.

## Materials and Methods

### Patient population

The Multi-omic Analytics and Integrative Neuroinformatics in the HUman Brain Lab at the UM has an approved and ongoing prospective TBI database for patients with moderate/severe TBI admitted to the intensive care unit (ICU) at Health Sciences Centre in Winnipeg (Shared Health, Manitoba, Canada; research ethics board [REB] approval # H2017:181, H2017:188, B2018:103, B2019:065, and B2023:001). The database was retrospectively reviewed for the purposes of this cohort study on existing archived data (REB approval # H2020:118, H2024:266). The selection criteria were adults who suffered moderate/severe TBI, requiring admission to the surgical ICU for invasive ICP monitoring. The data collected included patients’ demographics, admission injury data, computed tomographic injury patterns, treatments, and outcomes. In addition, all patients had high-frequency full waveform physiology collected, including ICP, ABP, and bifrontal rSO_2_. A total of 17 adult patients with moderate/severe TBI who received ketamine infusion were identified along with 105 who did not have ketamine infusions. Of the 17 patients who received ketamine, 13 patients had bifrontal rSO_2_ measurements.

### Signal acquisition and processing

Digital physiology data were captured at 0.1 Hz or higher from bedside monitoring devices using Intensive Care Monitoring “Plus” (ICM+, Cambridge Enterprise Ltd, Cambridge, UK, http://icmplus.neurosurg.cam.ac.uk), through direct digital output or analogue signal conversion (DT9804/DT9826, Data Translations, Marlboro, MA, USA). This platform captures all physiological data in a time-series format, allowing for future signal processing and analysis of the relationship between both raw and derived physiological parameters. ICP was acquired invasively through an intra-parenchymal strain gauge probe, and rSO_2_was measured via near-infrared spectroscopy regional oximetry of the left and right frontal lobes.

The following signal processing and analysis occurred using a similar methodology, covered in other publications from our lab.^23,24^ All signal processing was conducted using ICM+ and Python computing software. The pulse amplitude of ICP (AMP) was determined through Fourier analysis of the fundamental amplitude of the ICP pulse waveform.^25–27^

CA and compliance were assessed through various derived measures (COx/COx_a, PRx, PAx, RAC, and RAP), using standard methodologies for cerebral physiological signal processing in TBI.^27–30^ All indices were calculated in a similar fashion, using 30 consecutive 10-sec mean paired measures of input and output variables. Data for each patient were outputted in minute-by-minute comma separated value files for further processing, producing ICP, AMP, ABP, CPP, PRx, PAx, RAC, RAP, rSO_2_, and COx/COx_a. The definition of each of these indices can be found below:

- Pressure Reactivity (PRx): Correlation between ICP and ABP
- Pulse Amplitude (PAx): Correlation between AMP and ABP
- RAC: Correlation between AMP and CPP
- RAP: Correlation between AMP and ICP
- COx: Correlation between rSO_2_ and CPP
- COx_a: Correlation between rSO2 and ABP

To compare cerebral physiology within and between administration and dose changes of ketamine, we derived percentage time above/below clinically relevant thresholds for ICP,^3,31^ CPP,^3^ AMP,^27^ COx/COx_a,^28^ PRx,^31,32^ PAx,^32^ RAC,^32^ RAP,^25,27,33^ and rSO_2_.^21,22^ Prior to the analysis of ICP-derived indices, any ICP values greater than 50 mmHg or less than 0 mmHg were discarded. Similarly, prior to analysis of rSO_2_-derived indices, any data with rSO_2_ less than 0.1 was discarded. The evaluated threshold for each index can be found in Table 1.

**Table 1.** Evaluated Thresholds for Each Index

Index	Thresholds
ICP	>20 mmHg, >22 mmHg^3,31^
CPP	<60 mmHg, >70 mmHg^3,31^
PRx	>0, >0.25, >0.35^31,32^
PAx	>0, >0.25^32^
RAC	>0, >0.05^32^
RAP	>0, >0.40^25,27,33^
rSO_2_^a^	>60%, >70%, >80%, >90%^21,22^
COx/COx_a^a^	>0, >0.25^28,34,35^

^a^ Index evaluated for both left and right sides.

AMP, pulse amplitude of ICP; CPP, cerebral perfusion pressure; ICP, invasive intracranial pressure; RAC, correlation between AMP and CPP; RAP, correlation between AMP and ICP.

### Statistical analysis

All statistical analyses were conducted using Python 3.7 or R statistical packages. To assess for differences in patient demographics, Mann−Whitney *U* tests were performed for continuous variables and chi-squared test for categorical variables. An initial α was set to 0.05 and corrected for multiple comparisons using the Bonferroni methodology. Consequently, statistical significance was determined when *p* < 0.0029. Box plots, error-bar plots, and locally estimated scatterplot smoothing (LOESS) plots were utilized to describe and summarize the data. The statistical analysis was split into three components: (1) grand mean physiology compared between patients who received ketamine infusion and those who did not, (2) evaluation of mean hour physiology, and (3) evaluation of physiology surrounding each incremental dose change.

#### Grand mean analysis

To compare grand mean physiology between the two TBI patient groups (no ketamine, ketamine), mean index values and the percentage of time above/below clinically relevant thresholds were analyzed. These thresholds were defined based on current literature values, with the time above/below these limits found to be associated with worse long-term outcomes.^3,36^ Within the ketamine patient group, mean index value and percentage of time above/below thresholds were investigated for periods when the patient was receiving ketamine and when they were not, producing an “intrapatient” analysis. Box plots were used to visually describe the data and a Mann−Whitney *U* test was performed to assess statistical significance.

#### Evaluation of mean hourly physiology

To understand temporal trends in response to ketamine administration, the data from those patients with TBI receiving ketamine during their ICU stay were segmented into 1-h nonoverlapping time windows. If a window had less than 90% of its data, it was discarded from analysis. Mean index value versus mean ketamine infusion dose was compared. To demonstrate the data, a LOESS plot between the mean infusion dose and mean index value was created. The LOESS plot creates a trend line to help convey any response of cerebral physiology between the different infused doses. The infused doses were then binned into 11 equally segmented bins, with no ketamine being a bin of its own. The mean and 95% confidence intervals of each of these bins were then displayed on the LOESS plots.

#### Evaluation of each incremental dose change

In those patients with TBI receiving ketamine, we evaluated the physiology around each incremental dose change of ketamine. As such, four conditions of dose alterations were investigated:

1. Increase in infusion rate
2. Decrease in infusion rate
3. Going from “off” ketamine to “on” ketamine
4. Going from “on” ketamine to “off” ketamine

**FIG. 1.**
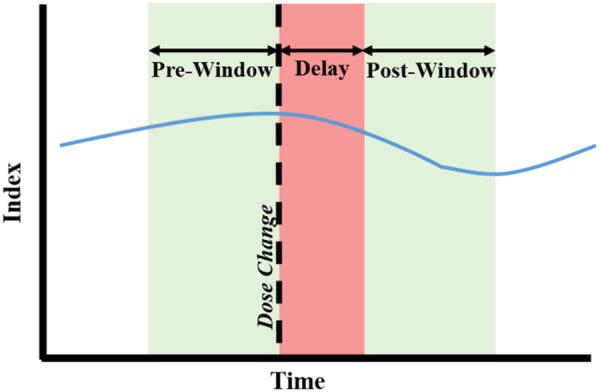
Graphical representation of windowing for incremental dose change analysis. Mean index was calculated in both the “pre-window” and “post-window.”

For each of these dose change conditions, data windows were analyzed immediately before the infusion rate change, and after the post infusion rate change, with a delay to account for the physiological response. Figure 1 shows a graphical representation of the time windows and delay. Various time windows were investigated ranging from 15 min to 2 h, and a delay of 15 min was chosen. Any time window with less than 90% of its data was discarded from analysis. Mean values were compared across these time windows, and a Mann−Whitney *U* test was performed. The variation in the time window did not lead to any significant differences. Thus, the time window and delay described in the remainder of the reported results within this article are 45 min and 15 min, respectively. Additionally, the percentage of time spent above/below clinical threshold was compared between time windows using the Mann−Whitney *U* test.

## Results

### Patient characteristics

Table 2 provides the patient characteristics for patients in the no ketamine and ketamine groups. There was no statistical difference found between any of the characteristics of each group. The median age was 37 (interquartile range [IQR]: 24–57) and 42 (IQR: 28–58) in the no ketamine and ketamine groups, respectively. The median Glasgow Coma Score motor subscore was 4 (IQR: 4–8) and 5 (IQR: 2–5) for the no ketamine and ketamine groups, respectively. Pre-hospital hypoxia, hypotension, pupillary light reflex, and Marshall CT score are all outlined in Table 2. Mann−Whitney *U* tests were performed for noncategorical variables, and chi-square tests were performed for categorical variables.

**Table 2.** Patient Characteristics

	Number (%) or median (IQR)		*p*-Value
	No ketamine	Ketamine	
Number of patients	105	17	—
Age	42 (28–58)	37 (24–57)	0.3869
Sex (male)	86 (81.9%)	15 (88.24%)	1.0
Best GCS	6 (4–8)	8 (4–8)	0.2222
Best GCS-motor	4 (2–5)	5 (2–5)	0.1680
Prehospital hypoxia	32 (30.48%)	8 (47.06%)	0.7966
Prehospital hypotension	12 (11.43 %)	1 (5.88%)	1.0
Pupillary light reflex:			0.4696
Bilateral reactive	60 (57.14%)	11 (64.71%)	—
Unilateral unreactive	26 (24.76%)	5 (29.41%)	—
Bilateral unreactive	17 (16.19%)	1 (5.88%)	—
Marshall CT Score:			0.7562
II	3 (2.86%)	0 (0%)	—
III	28 (26.67%)	6 (35.29%)	—
IV	16 (15.24%)	7 (41.18%)	—
V	58 (55.23%)	4 (23.53%)	—

GCS, Glasgow Coma Score; IQR, interquartile range.

### Grand mean analysis

#### Interpatient analysis

**FIG. 2.**
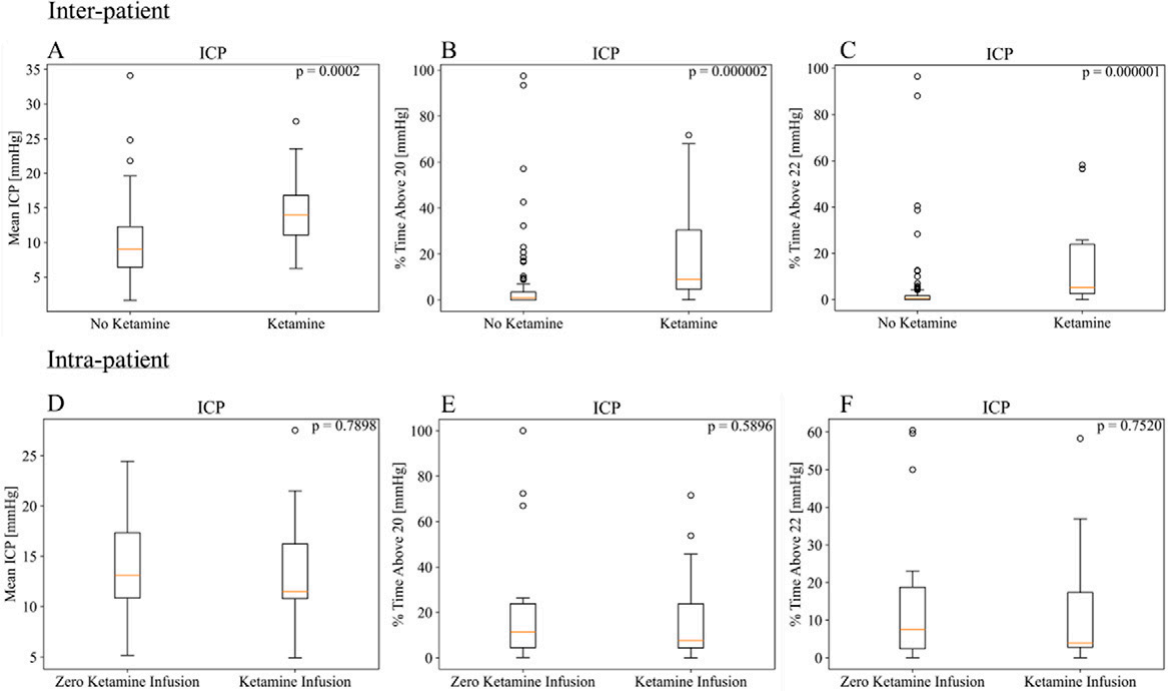
**(A–C)** Interpatient analysis between the no ketamine and ketamine groups: **(A)** Mean ICP. **(B)** Percentage time above ICP of 20 mmHg. **(C)** Percentage time above ICP of 22 mmHg. **(D–F)** Intrapatient analysis between zero ketamine infusion and ketamine infusion: **(D)** Mean ICP. **(E)** Percentage time above ICP of 20 mmHg **(C)** Percentage time above ICP of 22 mmHg. ICP, invasive intracranial pressure.

When comparing the mean index value between patients in the ketamine group and patients in the no ketamine group, a statistically significant difference was found in ICP (median: 9.05 mmHg and 14.00 mmHg, respectively, *p* = 0.00017). Similarly, a statistically significant difference in the percentage of time above threshold was observed between the no ketamine and ketamine groups for ICP at both threshold levels. The magnitude of difference between the percentage time above the ICP threshold for the no ketamine and ketamine group was small with the median ranging from 0.539% to 8.978%. Figure 2 illustrates these statistically significant differences found in ICP. No other statistically significant differences were observed in any other CVR index. All comparisons and their corresponding *p-*values can be found in Supplementary Tables S1 and S2of Supplementary Appendix SA1.

#### Intrapatient analysis

Within the ketamine group, periods of zero infusion rates were compared with periods of nonzero infusion rates. This analysis did not consider the magnitude of ketamine infusion. No significant associations were found in ICP, CPP, oxygen delivery, compliance, or any CVR index. Figure 2 compares the intrapatient and interpatient analysis of ICP. All comparisons and their corresponding *p-*values can be found in Supplementary Tables S3 and S4 of Supplementary Appendix SA1.

### Mean hourly physiology

**FIG. 3.**
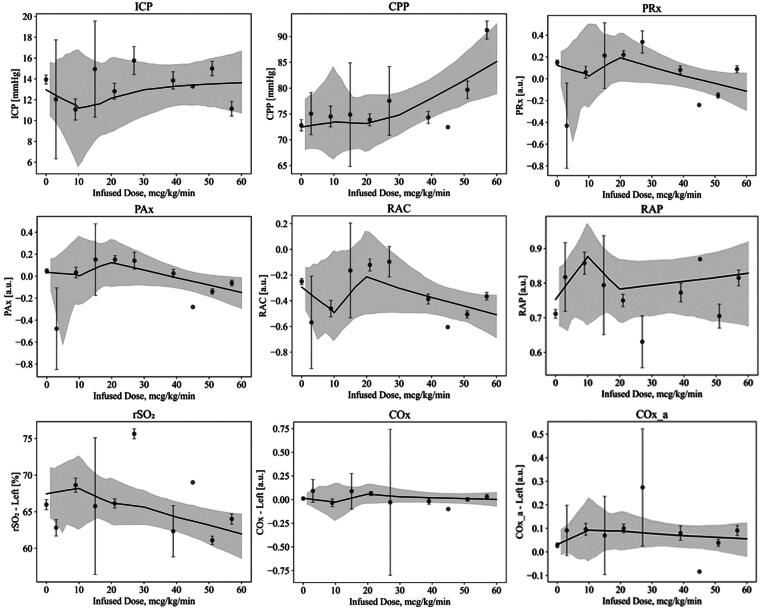
Mean hourly data for select indices of cerebral pressure-flow dynamics, illustrated with a LOESS curve with the 95% confidence interval. For rSO_2_, COx, and COx_a, only results for the left side are displayed. However, results for the right side showed similar trends. LOESS, locally estimated scatterplot smoothing; rSO_2_, regional cerebral oxygen saturation.

Within the ketamine group, the evaluation of mean hourly data showed no significant trends between the mean CVR indices and mean infused doses of ketamine. Specifically, there was no significant relationship between ketamine administration and ICP, CPP, oxygen delivery, compliance, or CVR measures. Figure 3 displays the LOESS curves for each index. For rSO_2_, COx, and COx_a, only the results for the left side are displayed; however, the results for the right side showed similar trends.

### Incremental dose change

**FIG. 4.**
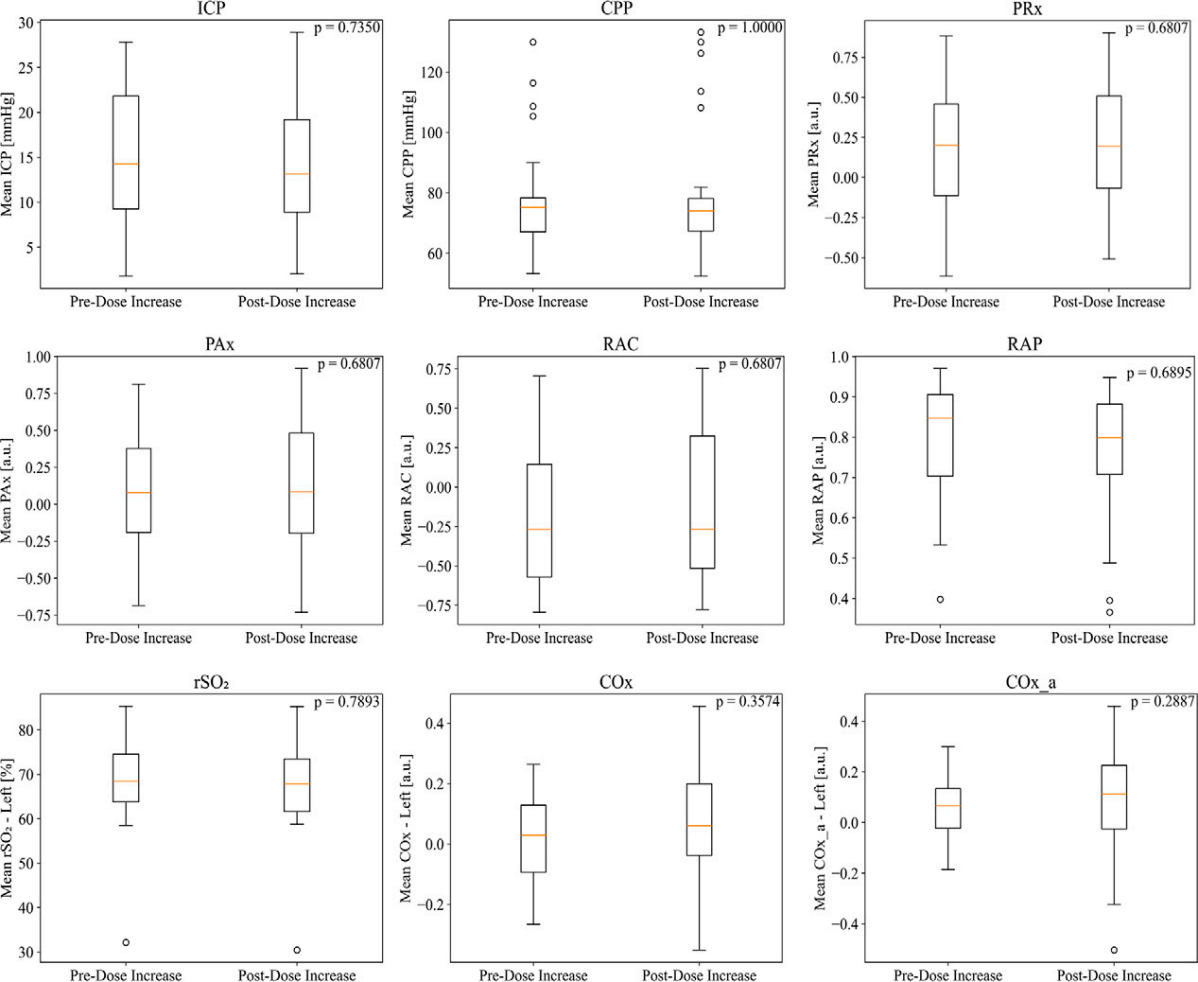
Mean index value pre- and post-infusion dose increase for select indices of cerebral pressure-flow dynamics. For rSO_2_, COx, and COx_a, only results for the left side are displayed. However, results for the right side showed similar trends. rSO_2_, regional cerebral oxygen saturation.

No significant trends were observed in ICP, CPP, oxygen delivery, compliance, or any CVR indices in response to all four incremental dose change conditions outlined above. Figure 4 demonstrates the comparison of mean index pre- and post-infusion increase for all the CVR indices. For rSO_2_, COx, and COx_a, only the results for the left side are displayed; however, results for the right side showed similar trends.

No statistically significant trends were found in infusion increases, infusion decreases, on-to-off transitions, or off-to-on transition conditions. All comparisons for each dose change condition, and their corresponding *p-*values can be found in Supplementary Tables S5 and S6 of Supplementary Appendix SA2.

## Discussion

Ketamine is an *N*-methyl-d-aspartate receptor antagonist known for its dissociative anesthesia, pain modulation, and vasoconstrictive properties.^37,38^ These benefits make ketamine an attractive anesthetic for use in patients with TBI. Despite these benefits, ketamine has largely been avoided in patients with TBI due to concerns of increased ICP, thought to be due to increased cerebral blood flow and increased cerebral metabolic rate for oxygen.^37^ More recent studies have demonstrated that ketamine does not increase ICP and may actually decrease it.^14,16^ Despite these findings, neurosurgery and neuroanesthesia literature continue to quote older articles that suggest ketamine elevates ICP.^10–13^ This literature lacks high-frequency physiological data and modern multimodal physiological monitoring. Therefore, the objective of this study was to investigate the impacts ketamine has on cerebral pressure-flow physiological measures by leveraging a unique high-resolution dataset.

When analyzing the impact of ketamine on ICP, a significant association was observed between patients who received ketamine and those who did not. The median mean ICP was higher in the ketamine group, approximately 14 mmHg, compared with approximately 9 mmHg in the no ketamine group. Additionally, the percentage of time above the thresholds for ICP was significantly different between the two groups, as seen in Figure 2. Since this was a retrospective analysis, it is possible that the patients received ketamine due to more severe secondary injury responses or were at risk of potential side effects from other sedative agents. This aligns with common clinical practice (including local practice) where ketamine is often introduced to reduce the risk of propofol infusion syndrome when a patient has been on propofol for an extended period. These findings are echoed by the lack of significant associations in the intrapatient analysis, mean hourly analysis, and across any incremental dose change conditions. Previous studies have demonstrated a decrease in ICP with administration of ketamine. However, these studies observed decreases with bolus administration of ketamine, whereas the patients in this study received ketamine infusions.^14–20^

When analyzing the impact of ketamine on CPP and mean arterial pressure (MAP), no significant findings were observed in the grand mean analysis, mean hourly physiology, or any incremental dose change condition. While nonsignificant, CPP and MAP appear to increase with increasing dose of ketamine; this is most likely due to the sympathomimetic effects that increase systemic ABP.

To assess ketamine’s impact on oxygen delivery, rSO_2_ was analyzed. No significant findings were observed from the grand mean analysis, mean hourly physiology, or any incremental dose change condition. A nonsignificant trend of decreasing rSO_2_ with increasing ketamine infusion was noted. A potential mechanism for this may be attributed to an increased cerebral metabolic rate for oxygen due to ketamine infusion.

CVR was evaluated by assessing PRx, PAx, RAC, COx, and COx_a. Negative or low values of these indices indicate good CVR/CA, while positive values suggest impaired CVR/CA. No significant findings were observed in the grand mean analysis, mean hourly physiology, or any incremental dose change condition. These results are consistent with a previous study that found no associations between PRx and ketamine infusions.^23^ Interestingly, the mean hourly physiology demonstrates a nonsignificant trend of decreasing CVR indices with an increase in ketamine infusion. Specifically, PRx appears to stay below a value of 0.2. It is known that a PRx greater than 0.2 is associated with poor long-term outcomes in patients with TBI.^39,40^ This decrease in PRx with increasing mean hourly ketamine dose may suggest that ketamine is increasing the systemic blood pressure, while ICP is remaining stable. Cerebrovascular compliance was also assessed via the RAP index; however, no significant findings were observed in the grand mean analysis, mean hourly physiology, or any incremental dose change condition.

### Limitations and future directions

While this dataset is globally unique, the small number of patients is a limitation to this study, and the above results are exploratory. The patient population is highly heterogeneous, and all patients’ cerebrovascular physiology would be influenced by individual patient characteristics, injury severity, and treatment regimen. The focus on basic statistics and general descriptive analysis provides a foundation for future more in-depth analyses. Future studies will need to incorporate a larger patient cohort with multivariable modeling to account for the complex patient characteristics and injury patterns in TBI populations.

## Conclusions

The results from this exploratory analysis showed that the administration of ketamine did not significantly impact measures of cerebral pressure-flow dynamics. The observed increase in mean ICP and the percentage time above threshold for ICP between the patients who received ketamine and those who did not is thought to be due to the difference in patient injury severity between the two groups. This idea was supported by the finding that there was no association between indices of cerebral pressure-flow dynamics and mean hourly or incremental dose changes of ketamine infusions. These exploratory results further contradict the historical concerns that ketamine elevates ICP. While further larger studies are needed, this study provides support for future studies into the potential benefits of ketamine in patients with TBI.

## Notes

Supplementary Appendix A1

Supplementary Appendix A2

## Abbreviations Used

ABP: Arterial Blood Pressure
AMP: Pulse Amplitude of ICP
CA: Cerebral Autoregulation
COx: Correlation between rSO2 and CPP COx_a - Correlation between
rSO2 and ABP CPP: Cerebral Perfusion Pressure
CVR: Cerebrovascular Reactivity
ICP: Intracranial Pressure
LOESS: Locally Estimated Scatterplot Smoothing
MAP: Mean Arterial Pressure
PAx: Pulse Amplitude (Correlation between AMP and ABP)
PRx: Pressure Reactivity (Correlation between ICP and ABP)
RAC: Correlation between AMP and CPP
RAP: Correlation between AMP and ICP
rSO2: Regional Cerebral Oxygen Saturation TBI - Traumatic Brain Injury

## References

[1] Maas AIR, Menon DK, Adelson PD, et al.; InTBIR Participants and Investigators. Traumatic brain injury: Integrated approaches to improve prevention, clinical care, and research. Lancet Neurol 2017;16(12):987–1048; doi: 10.1016/S1474-4422(17)30371-X 29122524

[2] Park E, Bell JD, Baker AJ. Traumatic brain injury: Can the consequences be stopped? CMAJ 2008;178(9):1163–1170; doi: 10.1503/cmaj.080282 18427091 PMC2292762

[3] Carney N, Totten AM, O’Reilly C, et al. Guidelines for the management of severe traumatic brain injury, fourth edition. Neurosurgery 2017;80(1):6–15; doi: 10.1227/NEU.0000000000001432 27654000

[4] Chesnut R, Aguilera S, Buki A, et al. A management algorithm for adult patients with both brain oxygen and intracranial pressure monitoring: The Seattle International Severe Traumatic Brain Injury Consensus Conference (SIBICC). Intensive Care Med 2020;46(5):919–929; doi: 10.1007/s00134-019-05900-x 31965267 PMC7210240

[5] Chesnut RM, Aguilera S, Buki A, et al. Perceived utility of intracranial pressure monitoring in traumatic brain injury: A seattle international brain injury consensus conference consensus-based analysis and recommendations. Neurosurgery 2023;93(2):399–408; doi: 10.1227/neu.0000000000002516 37171175 PMC10319366

[6] Hawryluk GWJ, Aguilera S, Buki A, et al. A management algorithm for patients with intracranial pressure monitoring: The Seattle International Severe Traumatic Brain Injury Consensus Conference (SIBICC). Intensive Care Med 2019;45(12):1783–1794; doi: 10.1007/s00134-019-05805-9 31659383 PMC6863785

[7] Sehdev RS, Symmons DAD, Kindl K. Ketamine for rapid sequence induction in patients with head injury in the emergency department. Emerg Med Australas 2006;18(1):37–44; doi: 10.1111/j.1742-6723.2006.00802.x 16454773

[8] Gregers MCT, Mikkelsen S, Lindvig KP, et al. Ketamine as an anesthetic for patients with acute brain injury: A systematic review. Neurocrit Care 2020;33(1):273–282; doi: 10.1007/s12028-020-00975-7 32328972 PMC7223585

[9] Himmelseher S, Durieux ME. Revising a dogma: Ketamine for patients with neurological injury? Anesth Analg 2005;101(2):524–534; doi: 10.1213/01.ANE.0000160585.43587.5B 16037171

[10] Wyte SR, Shapiro HM, Turner P, et al. Ketamine-induced intracranial hypertension. Anesthesiology 1972;36(2):174–176; doi: 10.1097/00000542-197202000-00021 5059108

[11] Shaprio HM, Wyte SR, Harris AB. Ketamine anaesthesia in patients with intracranial pathology. Br J Anaesth 1972;44(11):1200–1204; doi: 10.1093/bja/44.11.1200 4647115

[12] Gardner AE, Olson BE, Lichtiger M. Cerebrospinal-fluid pressure during dissociative anesthesia with ketamine. Anesthesiology 1971;35(2):226–228; doi: 10.1097/00000542-197108000-00029 5568142

[13] List WF, Crumrine RS, Cascorbi HF, et al. Increased cerebrospinal fluid pressure after ketamine. Anesthesiology 1972;36(1):98–99; doi: 10.1097/00000542-197201000-00023 5006995

[14] Bar-Joseph G, Guilburd Y, Tamir A, et al. Effectiveness of ketamine in decreasing intracranial pressure in children with intracranial hypertension. J Neurosurg Pediatr 2009;4(1):40–46; doi: 10.3171/2009.1.PEDS08319 19569909

[15] Bourgoin A, Albanèse J, Léone M, et al. Effects of sufentanil or ketamine administered in target-controlled infusion on the cerebral hemodynamics of severely brain-injured patients. Crit Care Med 2005;33(5):1109–1113; doi: 10.1097/01.ccm.0000162491.26292.98 15891344

[16] Albanese J, Arnaud S, Rey M, et al. Ketamine decreases intracranial pressure and electroencephalographic activity in traumatic brain injury patients during propofol sedation. Anesthesiology 1997;87(6):1328–1334; doi: 10.1097/00000542-199712000-00011 9416717

[17] Schmittner MD, Vajkoczy SL, Horn P, et al. Effects of fentanyl and S(+)-ketamine on cerebral hemodynamics, gastrointestinal motility, and need of vasopressors in patients with intracranial pathologies. J Neurosurg Anesthesiol 2007;19(4):257–262; doi: 10.1097/ANA.0b013e31811f3feb 17893578

[18] Zeiler FA, Teitelbaum J, West M, et al. The ketamine effect on ICP in traumatic brain injury. Neurocrit Care 2014;21(1):163–173; doi: 10.1007/s12028-013-9950-y 24515638

[19] Zeiler FA, Teitelbaum J, Gillman LM, et al. NMDA antagonists for refractory seizures. Neurocrit Care 2014;20(3):502–513; doi: 10.1007/s12028-013-9939-6 24519081

[20] Zeiler FA, Teitelbaum J, West M, et al. The ketamine effect on intracranial pressure in nontraumatic neurological illness. J Crit Care 2014;29(6):1096–1106; doi: 10.1016/j.jcrc.2014.05.024 24996763

[21] Mathieu F, Khellaf A, Ku JC, et al. Continuous near-infrared spectroscopy monitoring in adult traumatic brain injury: A systematic review. J Neurosurg Anesthesiol 2020;32(4):288–299; doi: 10.1097/ANA.0000000000000620 31306264

[22] Ng IHX, Da Costa CS, Zeiler FA, et al. Burden of hypoxia and intraventricular haemorrhage in extremely preterm infants. Arch Dis Child Fetal Neonatal Ed 2020;105(3):242–247; doi: 10.1136/archdischild-2019-316883 31256012

[23] Froese L, Dian J, Batson C, et al. The impact of vasopressor and sedative agents on cerebrovascular reactivity and compensatory reserve in traumatic brain injury: An exploratory analysis. Neurotrauma Rep 2020;1(1):157–168; doi: 10.1089/neur.2020.0028 33274344 PMC7703494

[24] Thelin EP, Raj R, Bellander B-M, et al. Comparison of high versus low frequency cerebral physiology for cerebrovascular reactivity assessment in traumatic brain injury: A multi-center pilot study. J Clin Monit Comput 2020;34(5):971–994; doi: 10.1007/s10877-019-00392-y 31573056 PMC7447671

[25] Calviello L, Donnelly J, Cardim D, et al. Compensatory-reserve-weighted intracranial pressure and its association with outcome after traumatic brain injury. Neurocrit Care 2018;28(2):212–220; doi: 10.1007/s12028-017-0475-7 29043546

[26] Froese L, Sainbhi AS, Gomez A, et al. Discrete fourier transform windowing techniques for cerebral physiological research in neural injury: A practical demonstration. Neurotrauma Rep 2023;4(1):410–419; doi: 10.1089/neur.2022.0079 37360544 PMC10288301

[27] Zeiler FA, Ercole A, Cabeleira M, et al.; CENTER-TBI High Resolution ICU Sub-Study Participants and Investigators. Compensatory-reserve-weighted intracranial pressure versus intracranial pressure for outcome association in adult traumatic brain injury: A CENTER-TBI validation study. Acta Neurochir (Wien) 2019;161(7):1275–1284; doi: 10.1007/s00701-019-03915-3 31053909 PMC6581920

[28] Brady KM, Lee JK, Kibler KK, et al. Continuous time-domain analysis of cerebrovascular autoregulation using near-infrared spectroscopy. Stroke 2007;38(10):2818–2825; doi: 10.1161/STROKEAHA.107.485706 17761921 PMC2377358

[29] Zeiler FA, Aries M, Czosnyka M, et al. Cerebral autoregulation monitoring in traumatic brain injury: An overview of recent advances in personalized medicine. J Neurotrauma 2022;39(21–22):1477–1494; doi: 10.1089/neu.2022.0217 35793108

[30] Zeiler FA, Ercole A, Czosnyka M, et al. Continuous cerebrovascular reactivity monitoring in moderate/severe traumatic brain injury: A narrative review of advances in neurocritical care. Br J Anaesth 2020;124(4):440–453; doi: 10.1016/j.bja.2019.11.031 31983411

[31] Sorrentino E, Diedler J, Kasprowicz M, et al. Critical thresholds for cerebrovascular reactivity after traumatic brain injury. Neurocrit Care 2012;16(2):258–266; doi: 10.1007/s12028-011-9630-8 21964774

[32] Zeiler FA, Donnelly J, Smielewski P, et al. Critical thresholds of ICP derived continuous cerebrovascular reactivity indices for outcome prediction in non-craniectomized TBI patients: PRx, PAx and RAC. J Neurotrauma 2018;35(10):1107–1115; doi: 10.1089/neu.2017.5472 29241396

[33] Zeiler FA, Kim D-J, Cabeleira M, et al. Impaired cerebral compensatory reserve is associated with admission imaging characteristics of diffuse insult in traumatic brain injury. Acta Neurochir (Wien) 2018;160(12):2277–2287; doi: 10.1007/s00701-018-3681-y 30251196 PMC6267721

[34] Laurikkala J, Aneman A, Peng A, et al. Association of deranged cerebrovascular reactivity with brain injury following cardiac arrest: A post-hoc analysis of the COMACARE trial. Crit Care 2021;25(1):350; doi: 10.1186/s13054-021-03764-6 34583763 PMC8477475

[35] Sainbhi AS, Froese L, Gomez A, et al. Continuous time-domain cerebrovascular reactivity metrics and discriminate capacity for the upper and lower limits of autoregulation: A scoping review of the animal literature. Neurotrauma Rep 2021;2(1):639–659; doi: 10.1089/neur.2021.0043 35018365 PMC8742280

[36] Lang EW, Kasprowicz M, Smielewski P, et al. Short pressure reactivity index versus long pressure reactivity index in the management of traumatic brain injury. J Neurosurg 2015;122(3):588–594; doi: 10.3171/2014.10.JNS14602 25423266

[37] Aroni F, Iacovidou N, Dontas I, et al. Pharmacological aspects and potential new clinical applications of ketamine: Reevaluation of an Old drug. J Clin Pharmacol 2009;49(8):957–964; doi: 10.1177/0091270009337941 19546251

[38] Visser E, Schug SA. The role of ketamine in pain management. Biomed Pharmacother 2006;60(7):341–348; doi: 10.1016/j.biopha.2006.06.021 16854557

[39] Lazaridis C, DeSantis SM, Smielewski P, et al. Patient-specific thresholds of intracranial pressure in severe traumatic brain injury: Clinical article. J Neurosurg 2014;120(4):893–900; doi: 10.3171/2014.1.JNS131292 24506248

[40] Zeiler FA, Ercole A, Cabeleira M, et al.; CENTER-TBI High Resolution ICU Sub-Study Participants and Investigators. Patient-specific ICP epidemiologic thresholds in adult traumatic brain injury: A CENTER-TBI validation study. J Neurosurg Anesthesiol 2021;33(1):28–38; doi: 10.1097/ANA.0000000000000616 31219937

