# Supplementary material for: High-Frequency Analysis of the Cerebral Physiological Impact of Ketamine in Acute Traumatic Neural Injury
Source: Neurotrauma Rep. 2025 Feb 20;6(1):232–41. doi: 10.1089/neur.2024.0146 (PMC11931103; doi:10.1089/neur.2024.0146)
Supplement: Supplementary Appendix A1 [file neur.2024.0146_supp_appendixa1.docx]

Appendix

Contents

[Legend 2](#_Toc176700127)

[Appendix A – Grand Mean Analysis 2](#_Toc176700128)

[Inter-Patient Analysis 2](#_Toc176700129)

[Intra-Patient Analysis 5](#_Toc176700130)

[Appendix B - Dose Change Analysis 8](#_Toc176700131)

[Infusion Increase 8](#_Toc176700132)

[Infusion Decrease 12](#_Toc176700133)

[On to Off 14](#_Toc176700134)

[Off to On 18](#_Toc176700135)

# Legend

**ICP:** Intracranial pressure

**CPP:** Cerebral perfusion pressure

**MAP:** Mean arterial pressure

**Pressure Reactivity (PRx):** Correlation between ICP and ABP

**Pulse Amplitude (PAx):** Correlation between AMP and ABP

**RAC:** Correlation between AMP and CPP

**RAP:** Correlation between AMP and ICP

**rSO_2_:** Regional cerebral oxygen saturation

**COx:** Correlation between rSO_2_ and CPP

**COx_a:** Correlation between rSO2 and ABP

# Appendix A – Grand Mean Analysis

## Inter-Patient Analysis

To compare grand mean physiology between the two TBI patient groups (no ketamine, ketamine), mean index values, and the percentage of time above/below clinically relevant thresholds outlined in the manuscript. To evaluate statistical significance Mann Whitney-U tests were performed.

1. Mean

Table I: Comparison of grand mean index between the no ketamine and ketamine group.

|  | **Median (IQR) – Mean Index** | |  |
| --- | --- | --- | --- |
| **Index** | **No Ketamine** | **Ketamine** | **P-Value** |
| **ICP [mmHg]** | **9.05 (6.43 – 12.28)** | **14.00 (11.08 – 16.81)** | **0.00017** |
| CPP  [mmHg] | 73.73 (69.64 – 78.45) | 72.83 (70.59 – 75.48) | 0.84892 |
| MAP  [mmHg] | 83.10 (79.02 – 87.86) | 87.78 (83.83 – 91.31) | 0.01082 |
| PRx | 0.1501 (0.0428 – 0.2765) | 0.0791 (0.0087 – 0.4467) | 0.98831 |
| PAx | 0.0133 (-0.1308 – 0.1170) | 0.0317 (-0.0891 – 0.3472) | 0.13047 |
| RAC | -0.2460 (-0.3847 – -0.0756) | -0.3676 (-0.4061 – 0.1114) | 0.87195 |
| RAP | 0.6188 (0.4900 – 0.7409) | 0.7359 (0.6965 – 0.8104) | 0.00805 |
| rSO_2__L  [%] | 70.82 (63.65 – 75.83) | 67.26 (63.49 – 69.71) | 0.29521 |
| rSO_2__R  [%] | 70.13 (64.12 – 77.97) | 69.38 (67.97 – 70.90) | 0.42420 |
| COx_a_L | 0.0757 (0.0263 – 0.1250) | 0.0568 (0.0314 – 0.0870) | 0.50751 |
| COx_a_R | 0.0580 (0.0090 – 0.1416) | 0.0425 (0.0289 – 0.1022) | 0.77668 |
| COx_L | 0.0272 (-0.0192 – 0.0876) | 0.0228 (0.0016 – 0.0517) | 0.92831 |
| COx_R | 0.0212 (-0.0371 – 0.1075) | 0.0175 (-0.0113 – 0.0515) | 0.71986 |

1. % Time

Table II: Comparison of percentage time above/below threshold between the no ketamine and ketamine group.

|  |  | **Median (IQR) - % Time Above/Below Threshold** | |  |
| --- | --- | --- | --- | --- |
| **Index** | **Threshold** | **No Ketamine [%]** | **Ketamine [%]** | **P-Value** |
| **ICP** | **22 [mmHg]** | **0.8786 (0.0** – **3.44)** | **8.978 (4.746** – **30.43)** | **1.86216E-06** |
|  | **20 [mmHg]** | **0.5392 (0.0** – **1.649)** | **5.195 (2.589** – **23.91)** | **5.79503E-07** |
| CPP | <60 [mmHg] | 3.756 (1.038 – 7.724) | 8.403 (2.929 – 12.74) | 0.09952 |
|  | 70 [mmHg] | 55.73 (37.16 – 72.89) | 60.91 (49.29 – 67.12) | 0.92995 |
| PRx | 0 [a.u.] | 64.66 (49.52 – 79.13) | 57.00 (41.87 – 70.70) | 0.49507 |
|  | 0.25 [a.u.] | 38.89 (24.56 – 55.47) | 31.12 (21.25 – 59.59) | 0.98247 |
|  | 0.35 [a.u.] | 28.80 (17.50 – 44.40) | 22.89 (15.91 – 58.09) | 0.96316 |
| PAx | 0 [a.u.] | 44.81 (30.94 – 62.98) | 54.72 (38.36 – 74.29) | 0.14638 |
|  | 0.25 [a.u.] | 21.01 (10.57 – 37.28) | 25.08 (16.71 – 55.24) | 0.07222 |
| RAC | 0 [a.u.] | 21.63 (11.42 – 38.26) | 18.08 (12.85 – 49.55) | 0.55249 |
|  | 0.05 [a.u.] | 18.78 (9.39 – 34.48) | 15.19 (10.82 – 47.62) | 0.46758 |
| RAP | 0 [a.u.] | 93.86 (88.43 – 96.36) | 95.92 (94.33 – 96.83) | 0.07840 |
|  | 0.4 [a.u.] | 78.71 (66.56 – 89.37) | 87.80 (84.13 – 92.29) | 0.02014 |
| rSO_2__L | 60 [%] | 96.50 (78.25 – 99.94) | 87.62 (74.05 – 95.70) | 0.38614 |
|  | 70 [%] | 50.75 (10.51 – 89.64) | 29.50 (11.78 – 48.79) | 0.47875 |
|  | 80 [%] | 3.165 (0.0000 – 24.98) | 1.109 (0.2299 – 4.978) | 0.51885 |
|  | 90 [%] | 0.0000 (0.0000 – 0.0774) | 0.0000 (0.0000 – 0.0139) | 0.87930 |
| rSO_2__R | 60 [%] | 96.94 (77.88 – 99.97) | 98.93 (90.85 – 100.0) | 0.51671 |
|  | 70 [%] | 53.08 (10.25 – 89.34) | 40.64 (31.47 – 60.65) | 0.41797 |
|  | 80 [%] | 7.111 (0.0000 – 28.72) | 0.6643 (0.0000 – 5.206) | 0.15610 |
|  | 90 [%] | 0.0000 (0.0000 – 1.426) | 0.0000 (0.0000 – 0.0000) | 0.10602 |
| COx_a_L | 0 [a.u.] | 52.37 (46.19 – 62.97) | 52.01 (47.12 – 58.79) | 0.71775 |
|  | 0.25 [a.u.] | 25.48 (20.10 – 32.91) | 26.13 (22.39 – 27.84) | 0.77330 |
| COx_a_R | 0 [a.u.] | 51.36 (44.54 – 60.87) | 50.96 (46.24 – 53.25) | 0.66452 |
|  | 0.25 [a.u.] | 25.49 (17.77 – 34.30) | 21.75 (19.43 – 26.33) | 0.32041 |
| COx_L | 0 [a.u.] | 46.66 (41.42 – 52.39) | 47.64 (42.87 – 49.92) | 0.79747 |
|  | 0.25 [a.u.] | 21.38 (16.97 – 28.56) | 23.23 (17.57 – 26.38) | 0.91244 |
| COx_R | 0 [a.u.] | 47.22 (37.42 – 56.74) | 44.87 (41.88 – 50.92) | 0.50978 |
|  | 0.25 [a.u.] | 21.90 (15.36 – 30.83) | 20.59 (18.52 – 22.86) | 0.45610 |

## Intra-Patient Analysis

Within the ketamine patient group, mean index value and percentage of time above thresholds were investigated for periods when the patient was receiving ketamine and when they were not, producing an “intra-patient” analysis.

1. Mean

Table III: Comparison of mean index between periods of ketamine infusion and periods of zero ketamine.

|  | **Median (IQR) – Mean Index** | |  |
| --- | --- | --- | --- |
| **Index** | **Zero Ketamine** | **Ketamine** | **P-Value** |
| ICP [mmHg] | 13.13 (10.86 – 17.35) | 11.51 (10.80 – 16.25) | 0.789843 |
| CPP  [mmHg] | 71.24 (67.00 – 76.13) | 74.25 (66.63 – 79.94) | 0.501947 |
| MAP  [mmHg] | 86.21 (80.24 – 90.26) | 85.77 (84.20 – 91.24) | 0.709547 |
| PRx | 0.1672 (0.0524 – 0.2541) | 0.0756 (0.0072 – 0.4990) | 0.709547 |
| PAx | 0.0171 (-0.0861 – 0.2295) | 0.0156 (-0.0905 – 0.4260) | 0.628085 |
| RAC | -0.297 (-0.3997 – -0.1249) | –0.4057 (-0.4635 – 0.2857) | 0.970308 |
| RAP | 0.6823 (0.6457 – 0.7740) | 0.7985 (0.6153 – 0.8520) | 0.094235 |
| rSO_2__L  [%] | 71.30 (65.52 – 76.51) | 65.46 (62.76 – 67.68) | 0.150667 |
| rSO_2__R  [%] | 73.32 (65.62 – 78.38) | 69.02 (67.47 – 70.20) | 0.150667 |
| COx_a_L | 0.0309 (-0.0051 – 0.0670) | 0.0914 (0.0583 – 0.1124) | 0.031744 |
| COx_a_R | 0.0244 (0.0018 – 0.0579) | 0.0718 (0.0250 – 0.1060) | 0.090842 |
| COx_L | 0.0229 (-0.0740 – 0.0458) | 0.0273 (-0.0105 – 0.1048) | 0.235117 |
| COx_R | -0.0134 (-0.0434 – 0.0083) | 0.0467 (-0.0019 – 0.0680 | 0.068817 |

1. %Time

Table IV: Comparison of percentage time above/below threshold between periods of ketamine infusion and periods of zero ketamine.

|  |  | **Median (IQR) - % Time Above Threshold** | |  |
| --- | --- | --- | --- | --- |
| **Index** | **Threshold** | **Zero Ketamine [%]** | **Ketamine [%]** | **P-Value** |
| ICP | 20 [mmHg] | 11.37 (4.520 – 23.78) | 7.568 (4.366 – 23.76) | 0.58958 |
|  | 22 [mmHg] | 7.577 (2.408 – 18.76) | 3.928 (2.809 – 17.38) | 0.75205 |
| CPP | <60 [mmHg] | 10.81 (3.539 – 23.96) | 2.715 (1.369 – 12.87) | 0.17126 |
|  | 70 [mmHg] | 47.14 (36.27 – 61.23) | 65.50 (46.09 – 84.09) | 0.13808 |
| PRx | 0 [a.u.] | 59.75 (51.50 – 70.05) | 54.10 (38.84 – 66.68) | 0.46767 |
|  | 0.25 [a.u.] | 39.02 (23.44 – 44.63) | 25.52 (17.73 – 49.11) | 0.61556 |
|  | 0.35 [a.u.] | 29.91 (15.81 – 40.03) | 17.58 (11.32 – 40.31) | 0.56410 |
| PAx | 0 [a.u.] | 49.01 (35.83 – 66.43) | 42.69 (36.62 – 85.25) | 0.69626 |
|  | 0.25 [a.u.] | 23.11 (15.39 – 50.77) | 20.07 (14.51 – 67.74) | 0.89653 |
| RAC | 0 [a.u.] | 23.29 (11.10 – 35.69) | 11.22 (8.351 – 67.20) | 0.83806 |
|  | 0.05 [a.u.] | 20.02 (9.836 – 32.22) | 9.077 (7.028 – 62.96) | 0.83806 |
| RAP | 0 [a.u.] | 93.75 (89.65 – 96.40) | 96.44 (94.81 – 97.80) | 0.10164 |
|  | 0.4 [a.u.] | 84.14 (75.40 – 88.89) | 91.60 (80.91 – 93.37) | 0.12817 |
| rSO_2__L | 60 [%] | 92.44 (79.31 – 99.88) | 86.62 (82.87 – 96.15) | 0.65219 |
|  | 70 [%] | 58.09 (16.29 – 84.07) | 18.76 (2.372 – 26.08) | 0.15128 |
|  | 80 [%] | 1.356 (0.2360 – 16.15) | 0.0000 (0.0000 – 0.4462) | 0.05567 |
|  | 90 [%] | 0.0000 (0.0000 – 0.0100) | 0.0000 (0.0000 – 0.0000) | 0.43846 |
| rSO_2__R | 60 [%] | 99.85 (91.90 – 100.0) | 98.06 (84.53 – 99.99) | 0.52885 |
|  | 70 [%] | 68.83 (14.54 – 96.77) | 25.90 (15.71 – 41.47) | 0.14314 |
|  | 80 [%] | 5.891 (0.0074 – 14.10) | 0.0000 (0.0000 – 0.5558) | 0.14314 |
|  | 90 [%] | 0.0000 (0.0000 – 0.0000) | 0.0000 (0.0000 – 0.0000) | 0.73936 |
| COx_a_L | 0 [a.u.] | 46.54 (42.62 – 56.31) | 53.40 (50.29 – 59.42) | 0.15127 |
|  | 0.25 [a.u.] | 22.62 (15.17 – 27.49) | 28.53 (25.17 – 30.81) | 0.07589 |
| COx_a_R | 0 [a.u.] | 48.55 (41.63 – 52.30) | 54.70 (49.42 – 59.84) | 0.14314 |
|  | 0.25 [a.u.] | 19.85 (15.57 – 23.34) | 26.85 (19.52 – 28.70) | 0.16549 |
| COx_L | 0 [a.u.] | 43.98 (33.71 – 51.64) | 48.67 (43.08 – 50.79) | 0.56189 |
|  | 0.25 [a.u.] | 22.11 (13.92 – 25.83) | 22.11 (19.14 – 28.89) | 0.40092 |
| COx_R | 0 [a.u.] | 38.31 (33.45 – 46.52) | 51.83 (31.51 – 52.23) | 0.43587 |
|  | 0.25 [a.u.] | 15.41 (14.22 – 21.87) | 20.82 (12.84 – 27.02) | 0.52885 |
