# Supplementary material for: High-Frequency Analysis of the Cerebral Physiological Impact of Ketamine in Acute Traumatic Neural Injury
Source: Neurotrauma Rep. 2025 Feb 20;6(1):232–41. doi: 10.1089/neur.2024.0146 (PMC11931103; doi:10.1089/neur.2024.0146)
Supplement: Supplementary Appendix A2 [file neur.2024.0146_supp_appendixa2.docx]

# Appendix B - Dose Change Analysis

The physiology around each incremental dose change of ketamine was analyzed for the TBI patients receiving ketamine. As such, four conditions of dose alterations were investigated:

- Increase in infusion rate
- Decrease in infusion rate
- Going from “off” ketamine to “on” ketamine
- Going from “on” ketamine to “off” ketamine

A description of the methodology used to compare around each dose condition can be found in the associated manuscript. To evaluate statistical significance Mann Whitney-U tests were performed.

## Infusion Increase

1. Median

Table V: Comparison of grand mean index pre- and post -dose infusion increase.

|  | **Median (IQR) – Mean Index** | |  |
| --- | --- | --- | --- |
| **Index** | **Pre-Dose Change** | **Post-Dose Change** | **P-Value** |
| ICP [mmHg] | 14.26 (9.268 – 21.82) | 13.15 (8.904 – 19.20) | 0.734975 |
| CPP  [mmHg] | 75.19 (67.10 – 78.34) | 74.04 (67.37 – 78.16) | 1 |
| MAP  [mmHg] | 88.92 (80.62 – 94.92) | 87.78 (80.28 – 93.14) | 0.91635 |
| PRx | 0.1996 (-0.1133 – 0.4576) | 0.1968 (-0.0662 – 0.5082) | 0.680696 |
| PAx | 0.0801 (-0.1900 – 0.3775) | 0.0844 (-0.1964 – 0.4818) | 0.680696 |
| RAC | -0.2690 (-0.5705 – 0.1450) | –0.2682 (-0.5150 – 0.3245) | 0.680696 |
| RAP | 0.8474 (0.7034 – 0.9058) | 0.7992 (0.7076 – 0.8818) | 0.689468 |
| rSO_2__L  [%] | 68.44 (63.81 – 74.54) | 67.89 (61.64 – 73.47) | 0.789307 |
| rSO_2__R  [%] | 71.34 (68.28 – 74.18) | 69.47 (68.47 – 73.29) | 0.765173 |
| COx_a_L | 0.0660 (-0.0228 – 0.1343) | 0.1118 (-0.0256 – 0.2258) | 0.28866 |
| COx_a_R | 0.1161 (-0.0312 – 0.1536) | 0.0403 (-0.0245 – 0.1609) | 0.651481 |
| COx_L | 0.0292 (-0.0935 – 0.1287) | 0.0608 (-0.0377 – 0.1997) | 0.357437 |
| COx_R | 0.0066 (-0.0488 – 0.1049) | 0.0459 (-0.0386 – 0.1947) | 0.400663 |

1. %Time

Table VI: Comparison of percentage time above/below threshold index pre- and post -dose infusion increase.

|  |  | **Median (IQR) - % Time Above Threshold** | |  |
| --- | --- | --- | --- | --- |
| **Index** | **Threshold** | **Pre-Dose Change [%]** | **Post-Dose Change [%]** | **P-Value** |
| ICP | 20 [mmHg] | 0.000 (0.000 – 0.000) | 0.000 (0.000 – 14.71) | 0.54986 |
|  | 22 [mmHg] | 0.000 (0.000 – 0.000) | 0.000 (0.000 – 10.87) | 0.42482 |
| CPP | <60[mmHg] | 0.000 (0.000 – 6.522) | 0.000 (0.000 – 8.696) | 0.84219 |
|  | 70 [mmHg] | 80.43 (14.63 – 100.0) | 67.39 (34.78 – 91.30) | 0.50098 |
| PRx | 0 [a.u.] | 39.13 (13.04 – 78.26) | 47.83 (19.35 – 76.19) | 0.45446 |
|  | 0.25 [a.u.] | 8.696 (4.348 – 65.22) | 31.11 (13.33 – 60.87) | 0.19296 |
|  | 0.35 [a.u.] | 4.348 (0.000 – 63.04) | 24.44 (6.522 – 50.00) | 0.17618 |
| PAx | 0 [a.u.] | 39.13 (9.756 – 65.22) | 41.30 (15.22 – 90.70) | 0.43950 |
|  | 0.25 [a.u.] | 10.87 (2.174 – 56.52) | 17.78 (4.444 – 61.76) | 0.50098 |
| RAC | 0 [a.u.] | 6.522 (0.000 – 43.48) | 15.56 (2.174 – 50.00) | 0.39631 |
|  | 0.05 [a.u.] | 6.522 (0.000 – 39.13) | 11.11 (2.174 – 47.83) | 0.36897 |
| RAP | 0 [a.u.] | 100.0 (93.48 – 100.0) | 97.06 (91.11 – 100.0) | 0.51702 |
|  | 0.4 [a.u.] | 100.0 (91.30 – 100.0) | 91.30 (86.96 – 100.0) | 0.46970 |
| rSO_2__L | 60 [%] | 100.0 (88.89 – 100.0) | 100.0 (83.87 – 100.0) | 0.60093 |
|  | 70 [%] | 23.91 (0.000 – 100.0) | 11.11 (0.000 – 95.65) | 0.88131 |
|  | 80 [%] | 0.000 (0.000 – 0.000) | 0.000 (0.000 – 0.000) | 0.96031 |
|  | 90 [%] | 0.000 (0.000 – 0.000) | 0.000 (0.000 – 0.000) | 1.00000 |
| rSO_2__R | 60 [%] | 100.0 (100.0 – 100.0) | 100.0 (94.12 – 100.0) | 0.18443 |
|  | 70 [%] | 58.70 (0.000 – 100.0) | 4.651 (0.000 – 97.83) | 0.56665 |
|  | 80 [%] | 0.000 (0.000 – 0.000) | 0.000 (0.000 – 0.000) | 0.98015 |
|  | 90 [%] | 0.000 (0.000 – 0.000) | 0.000 (0.000 – 0.000) | 1.00000 |
| COx_a_L | 0 [a.u.] | 56.52 (39.13 – 65.22) | 55.88 (32.61 – 73.91) | 0.60093 |
|  | 0.25 [a.u.] | 21.74 (10.87 – 32.61) | 17.39 (11.63 – 43.48) | 0.54986 |
| COx_a_R | 0 [a.u.] | 54.35 (36.96 – 69.57) | 54.35 (39.13 – 60.87) | 0.65399 |
|  | 0.25 [a.u.] | 26.09 (8.696 – 34.78) | 17.39 (6.977 – 32.61) | 0.69037 |
| COx_L | 0 [a.u.] | 43.48 (32.61 – 63.04) | 51.61 (32.61 – 67.44) | 0.43950 |
|  | 0.25 [a.u.] | 15.22 (8.696 – 34.78) | 17.39 (8.696 – 47.83) | 0.53332 |
| COx_R | 0 [a.u.] | 43.48 (30.43 – 56.10) | 47.83 (24.44 – 64.44) | 0.60093 |
|  | 0.25 [a.u.] | 21.74 (6.522 – 30.43) | 15.22 (2.174 – 36.96) | 0.98015 |

## Infusion Decrease

1. Median

Table VII: Comparison of grand mean index pre- and post -dose infusion decrease.

|  | **Median (IQR) – Mean Index** | |  |
| --- | --- | --- | --- |
| **Index** | **Pre-Dose Change** | **Post-Dose Change** | **P-Value** |
| ICP [mmHg] | 8.8803(6.2259-12.4939) | 9.2842(6.2599-11.8445) | 0.76922 |
| CPP  [mmHg] | 81.23(76.0329-88.4398) | 81.1588(68.1824-89.7368) | 0.68113 |
| MAP  [mmHg] | 87.3872(81.8567-104.9348) | 88.0493(74.7172-101.9764) | 0.73947 |
| PRx | 0.0395(-0.1037-0.2351) | 0.1553(-0.0419-0.1944) | 0.55210 |
| PAx | -0.0783(-0.1745-0.036) | -0.0799(-0.2344-0.0653) | 0.95103 |
| RAC | -0.4166(-0.6365--0.1708) | -0.4715(-0.6544--0.1407) | 0.85383 |
| RAP | 0.8318(0.711-0.9284) | 0.8137(0.7351-0.9274) | 0.88606 |
| rSO_2__L  [%] | 69.5013(66.0434-73.4921) | 70.8109(69.0402-72.353) | 1.00000 |
| rSO_2__R  [%] | 69.2076(67.4389-71.9798 | 69.6433(69.0329-72.4061) | 0.70450 |
| COx_a_L | 0.085(0.0043-0.2483) | 0.085(0.031-0.1533) | 0.45610 |
| COx_a_R | 0.0573(0.0049-0.3274) | 0.0251(-0.0634-0.1593) | 0.35623 |
| COx_L | 0.0399(-0.0477-0.2913) | -0.019(-0.1103-0.1269 | 0.40018 |
| COx_R | -0.1058(-0.1132-0.3088) | -0.0806(-0.1076--0.0123) | 0.90483 |

1. %Time

Table VIII: Comparison of percentage time above/below threshold index pre- and post -dose infusion decrease.

|  |  | **Median (IQR) - % Time Above Threshold** | |  |
| --- | --- | --- | --- | --- |
| **Index** | **Threshold** | **Pre-Dose Change [%]** | **Post-Dose Change [%]** | **P-Value** |
| ICP | 20 [mmHg] | 0.0000 (0.0000 - 0.0000) | 0.0000 (0.0000 - 0.0000) | 0.840385 |
|  | 22 [mmHg] | 0.0000 (0.0000 - 0.0000) | 0.0000 (0.0000 - 0.0000) | 0.840385 |
| CPP | <60 [mmHg] | 0.0000 (0.0000 - 4.348) | 0.000 (0.000 - 0.000) | 0.544759 |
|  | 70 [mmHg] | 100.0 (42.39 - 100.0) | 84.78 (21.27 - 100.0) | 0.599878 |
| PRx | 0 [a.u.] | 52.94 (34.78 - 85.04) | 70.39 (42.39 - 78.26) | 0.840385 |
|  | 0.25 [a.u.] | 35.29 (9.783 - 46.69) | 33.00 (22.28 - 38.04) | 1 |
|  | 0.35 [a.u.] | 17.65 (5.435 - 32.21) | 21.74 (16.59 - 28.80) | 0.657405 |
| PAx | 0 [a.u.] | 32.61 (23.51 - 51.00) | 36.96 (24.80 - 46.38) | 0.903892 |
|  | 0.25 [a.u.] | 8.696 (3.261 - 35.36) | 11.96 (7.685 - 20.11) | 0.657405 |
| RAC | 0 [a.u.] | 6.522 (0.000 - 22.83) | 3.333 (0.000 - 20.36) | 0.967849 |
|  | 0.05 [a.u.] | 2.174 (0.000 - 17.39) | 3.333 (0.000 - 16.44) | 0.903892 |
| RAP | 0 [a.u.] | 97.83 (78.26 - 100.0) | 98.89 (94.57 - 100.0) | 0.544759 |
|  | 0.4 [a.u.] | 86.96 (45.21 - 98.91) | 91.30 (77.39 - 98.37) | 0.716784 |
| rSO_2__L | 60 [%] | 100.0 (92.39 - 100.0) | 100.0 (100.0 - 100.0) | 0.492022 |
|  | 70 [%] | 26.09 (13.04 - 100.0) | 64.13 (17.39 - 95.11) | 0.967849 |
|  | 80 [%] | 0.000 (0.000 - 2.250) | 0.000 (0.000 - 0.5556) | 0.657405 |
|  | 90 [%] | 0.000 (0.000 - 0.000) | 0.000 (0.000 - 0.000) | 1 |
| rSO_2__R | 60 [%] | 100.0 (86.43 - 100.0) | 100.0 (83.44 - 100.0) | 0.967849 |
|  | 70 [%] | 18.18 (4.348 - 42.39) | 12.03 (3.261 - 45.93) | 0.840385 |
|  | 80 [%] | 0.000 (0.000 - 0.000) | 0.000 (0.000 - 0.000) | 0.967849 |
|  | 90 [%] | 0.000 (0.000 - 0.000) | 0.000 (0.000 - 0.000) | 1 |
| COx_a_L | 0 [a.u.] | 63.04 (56.52 - 89.10) | 56.52 (53.26 - 64.72) | 0.310047 |
|  | 0.25 [a.u.] | 32.61 (22.83 - 54.78) | 29.35 (18.48 - 37.20) | 0.492022 |
| COx_a_R | 0 [a.u.] | 52.17 (48.91 - 71.17) | 48.91 (42.39 - 66.11) | 0.544759 |
|  | 0.25 [a.u.] | 28.26 (16.30 - 45.65) | 24.90 (18.48 - 34.78) | 0.903892 |
| COx_L | 0 [a.u.] | 47.83 (36.96 - 73.57) | 39.13 (32.07 - 70.78) | 0.657405 |
|  | 0.25 [a.u.] | 19.57 (15.22 - 56.17) | 21.74 (17.14 - 37.50) | 0.777989 |
| COx_R | 0 [a.u.] | 39.13 (25.00 - 63.08) | 34.01 (28.26 - 45.40) | 0.777989 |
|  | 0.25 [a.u.] | 17.39 (13.04 - 43.55) | 14.30 (8.152 - 22.81) | 0.492022 |

## On to Off

1. Median

Table IX: Comparison of grand mean index for “on to off”.

|  | **Median (IQR) – Mean Index** | |  |
| --- | --- | --- | --- |
| **Index** | **Pre-Dose Change** | **Post-Dose Change** | **P-Value** |
| ICP [mmHg] | 12.34 (7.295 - 14.06) | 11.76 (9.074 - 11.91) | 0.502727 |
| CPP  [mmHg] | 86.76 (79.46 - 102.9) | 81.16 (69.11 - 113.3) | 0.551631 |
| MAP  [mmHg] | 94.84 (86.69 - 118.1) | 101.0 (74.19 - 125.0) | 0.655632 |
| PRx | 0.0551 (-0.1197 - 0.1475) | 0.1509 (-0.1324 - 0.2005) | 0.602679 |
| PAx | -0.0605 (-0.2234 - 0.1588) | -0.0605 (-0.3090 - 0.2125) | 0.823756 |
| RAC | -0.5362 (-0.6472 - -0.1687) | -0.5052 (-0.7156 - -0.1131) | 0.881984 |
| RAP | 0.8385 (0.7401 - 0.9190) | 0.8908 (0.7313 - 0.9336) | 0.940843 |
| rSO_2__L  [%] | 69.50 (62.97 - 72.95) | 65.86 (59.08 - 70.87) | 0.555556 |
| rSO_2__R  [%] | 69.77 (69.21 - 74.13) | 71.04 (63.81 - 73.12) | 1 |
| COx_a_L | 0.0850 (0.0555 - 0.1782) | -0.0155 (-0.0773 - 0.0616) | 0.190476 |
| COx_a_R | 0.1190 (0.0442 - 0.2541) | -0.0780 (-0.0870 - -0.0431) | 0.057143 |
| COx_L | 0.0336 (-0.0190 - 0.2445) | 0.0281 (-0.0857 - 0.1454) | 0.412698 |
| COx_R | 0.0515 (-0.1512 - 0.2833) | -0.0916 (-0.1125 - -0.0816) | 0.885714 |

1. %Time

Table X: Comparison of percentage time above/below threshold index for “on to off” .

|  |  | **Median (IQR) - % Time Above Threshold** | |  |
| --- | --- | --- | --- | --- |
| **Index** | **Threshold** | **Pre-Dose Change [%]** | **Post-Dose Change [%]** | **P-Value** |
| ICP | 20 [mmHg] | 0.0000 (0.0000 - 0.0000) | 0.0000 (0.0000 - 3.261) | 1 |
|  | 22 [mmHg] | 0.0000 (0.0000 - 0.0000) | 0.0000 (0.0000 - 3.261) | 1 |
| CPP | <60 [mmHg] | 0.0000 (0.0000 - 0.0000) | 0.0000 (0.0000 - 1.087) | 0.571429 |
|  | 70 [mmHg] | 100.0 (100.0 - 100.0) | 69.57 (41.30 - 84.78) | 0.392857 |
| PRx | 0 [a.u.] | 82.61 (52.94 - 83.72) | 78.26 (52.17 - 78.26) | 0.392857 |
|  | 0.25 [a.u.] | 41.30 (35.29 - 45.65) | 30.43 (27.17 - 35.87) | 0.571429 |
|  | 0.35 [a.u.] | 21.74 (17.65 - 32.61) | 21.74 (21.74 - 25.00) | 1 |
| PAx | 0 [a.u.] | 58.82 (26.09 - 80.43) | 52.17 (34.78 - 73.91) | 0.785714 |
|  | 0.25 [a.u.] | 41.18 (8.696 - 45.65) | 15.22 (14.13 - 25.00) | 0.785714 |
| RAC | 0 [a.u.] | 16.28 (0.0000 - 17.39) | 0.0000 (0.0000 - 16.30) | 0.785714 |
|  | 0.05 [a.u.] | 13.95 (0.0000 - 15.22) | 0.0000 (0.0000 - 11.96) | 0.785714 |
| RAP | 0 [a.u.] | 100.0 (100.0 - 100.0) | 100.0 (95.65 - 100.0) | 1 |
|  | 0.4 [a.u.] | 100.0 (76.47 - 100.0) | 93.48 (81.52 - 96.74) | 1 |
| rSO_2__L | 60 [%] | 100.0 (89.13 - 100.0) | 100.0 (50.00 - 100.0) | 1 |
|  | 70 [%] | 19.57 (13.04 - 52.94) | 52.17 (26.09 - 64.13) | 1 |
|  | 80 [%] | 0.0000 (0.0000 - 2.326) | 0.0000 (0.0000 - 0.0000) | 0.571429 |
|  | 90 [%] | 0.0000 (0.0000 - 0.0000) | 0.0000 (0.0000 - 0.0000) | 1 |
| rSO_2__R | 60 [%] | 89.13 (83.72 - 100.0) | 100.0 (50.00 - 100.0) | 1 |
|  | 70 [%] | 27.91 (4.348 - 43.48) | 17.39 (8.696 - 58.70) | 1 |
|  | 80 [%] | 0.0000 (0.0000 - 0.0000) | 0.0000 (0.0000 - 0.0000) | 1 |
|  | 90 [%] | 0.0000 (0.0000 - 0.0000) | 0.0000 (0.0000 - 0.0000) | 1 |
| COx_a_L | 0 [a.u.] | 63.04 (58.70 - 69.77) | 50.00 (41.30 - 56.52) | 0.25 |
|  | 0.25 [a.u.] | 41.30 (28.26 - 46.51) | 8.696 (7.609 - 21.74) | 0.142857 |
| COx_a_R | 0 [a.u.] | 58.70 (52.17 - 62.79) | 43.48 (38.04 - 46.74) | 0.071429 |
|  | 0.25 [a.u.] | 30.23 (28.26 - 32.61) | 19.57 (13.04 - 26.09) | 0.392857 |
| COx_L | 0 [a.u.] | 56.52 (39.13 - 74.42) | 69.57 (51.09 - 72.83) | 1 |
|  | 0.25 [a.u.] | 19.57 (19.57 - 55.81) | 23.91 (16.30 - 38.04) | 0.785714 |
| COx_R | 0 [a.u.] | 39.13 (32.61 - 51.16) | 28.26 (27.17 - 35.87) | 0.571429 |
|  | 0.25 [a.u.] | 17.39 (13.04 - 32.56) | 8.696 (4.348 - 14.13) | 0.571429 |

## Off to On

1. Median

Table XI: Comparison of grand mean index for “off to on”.

|  | **Median (IQR) – Mean Index** | |  |
| --- | --- | --- | --- |
| **Index** | **Pre-Dose Change** | **Post-Dose Change** | **P-Value** |
| ICP [mmHg] | 14.89 (8.360 - 21.21) | 12.60 (8.117 - 17.80) | 0.719269 |
| CPP  [mmHg] | 74.83 (67.01 - 88.36) | 75.87 (67.95 - 101.6) | 0.742798 |
| MAP  [mmHg] | 87.30 (79.35 - 105.8) | 87.90 (80.26 - 116.2) | 0.742798 |
| PRx | 0.0453 (-0.1295 - 0.2948) | 0.1464 (-0.0727 - 0.4368) | 0.613231 |
| PAx | -0.0482 (-0.1964 - 0.3024) | -0.0165 (-0.2212 - 0.3364) | 0.883447 |
| RAC | -0.3686 (-0.6102 - -0.0417) | -0.3494 (-0.5581 - 0.0329) | 0.613231 |
| RAP | 0.8490 (0.7160 - 0.9094) | 0.7963 (0.6967 - 0.8971) | 0.659844 |
| rSO_2__L  [%] | 67.07 (63.96 - 75.73) | 66.79 (62.41 - 73.94) | 0.747658 |
| rSO_2__R  [%] | 72.06 (65.41 - 76.74) | 71.79 (67.21 - 75.43) | 0.853428 |
| COx_a_L | 0.0970 (-0.0520 - 0.1069) | 0.1609 (0.0932 - 0.2153) | 0.087946 |
| COx_a_R | 0.0560 (-0.0580 - 0.1429) | 0.0942 (-0.0066 - 0.1871) | 0.48251 |
| COx_L | -0.0117 (-0.1117 - 0.0876) | 0.1395 (0.0069 - 0.1996) | 0.065169 |
| COx_R | -0.0499 (-0.1158 - 0.0476) | 0.0530 (-0.0315 - 0.2141) | 0.247451 |

1. %Time

Table XII: Comparison of percentage time above/below threshold index for “off to on”.

|  |  | **Median (IQR) - % Time Above Threshold** | |  |
| --- | --- | --- | --- | --- |
| **Index** | **Threshold** | **Pre-Dose Change [%]** | **Post-Dose Change [%]** | **P-Value** |
| ICP | 20 [mmHg] | 0.0000 (0.0000 - 39.13) | 7.353 (0.0000 - 30.43) | 0.739364 |
|  | 22 [mmHg] | 0.0000 (0.0000 - 35.87) | 5.435 (0.0000 - 10.87) | 0.739364 |
| CPP | <60 [mmHg] | 0.0000 (0.0000 - 5.435) | 1.087 (0.0000 - 15.41) | 0.739364 |
|  | 70 [mmHg] | 42.63 (5.289 - 86.66) | 72.83 (39.32 - 97.56) | 0.578742 |
| PRx | 0 [a.u.] | 33.20 (24.46 - 44.54) | 51.85 (21.58 - 69.57) | 0.352681 |
|  | 0.25 [a.u.] | 8.696 (3.344 - 11.96) | 30.43 (8.696 - 58.15) | 0.190316 |
|  | 0.35 [a.u.] | 2.735 (0.0000 - 5.978) | 25.00 (6.224 - 50.00) | 0.123005 |
| PAx | 0 [a.u.] | 21.74 (11.66 - 39.33) | 35.87 (17.86 - 72.83) | 0.352681 |
|  | 0.25 [a.u.] | 5.700 (2.717 - 12.81) | 14.13 (2.419 - 55.02) | 0.528849 |
| RAC | 0 [a.u.] | 3.526 (0.0000 - 9.783) | 9.187 (0.0000 - 38.04) | 0.630529 |
|  | 0.05 [a.u.] | 2.439 (0.0000 - 6.522) | 8.100 (0.0000 - 34.78) | 0.481251 |
| RAP | 0 [a.u.] | 95.71 (89.13 - 100.0) | 98.53 (86.96 - 100.0) | 0.853428 |
|  | 0.4 [a.u.] | 95.71 (79.03 - 100.0) | 91.24 (83.70 - 100.0) | 0.911797 |
| rSO_2__L | 60 [%] | 100.0 (91.67 - 100.0) | 100.0 (87.34 - 100.0) | 0.795936 |
|  | 70 [%] | 11.96 (0.0000 - 97.22) | 5.435 (0.0000 - 96.32) | 0.970512 |
|  | 80 [%] | 0.0000 (0.0000 - 0.0000) | 0.0000 (0.0000 - 0.0000) | 0.970512 |
|  | 90 [%] | 0.0000 (0.0000 - 0.0000) | 0.0000 (0.0000 - 0.0000) | 1 |
| rSO_2__R | 60 [%] | 100.0 (100.0 - 100.0) | 100.0 (87.12 - 100.0) | 0.578742 |
|  | 70 [%] | 50.00 (0.0000 - 100.0) | 51.09 (0.0000 - 100.0) | 1 |
|  | 80 [%] | 0.0000 (0.0000 - 0.0000) | 0.0000 (0.0000 - 0.0000) | 1 |
|  | 90 [%] | 0.0000 (0.0000 - 0.0000) | 0.0000 (0.0000 - 0.0000) | 1 |
| COx_a_L | 0 [a.u.] | 41.30 (30.98 - 65.24) | 61.96 (38.43 - 78.42) | 0.279861 |
|  | 0.25 [a.u.] | 16.70 (11.41 - 21.90) | 27.03 (12.50 - 41.85) | 0.314999 |
| COx_a_R | 0 [a.u.] | 39.13 (33.15 - 64.06) | 48.14 (16.30 - 60.87) | 0.795936 |
|  | 0.25 [a.u.] | 16.30 (5.435 - 29.76) | 16.30 (2.437 - 31.52) | 1 |
| COx_L | 0 [a.u.] | 33.70 (27.52 - 44.54) | 57.29 (35.75 - 64.13) | 0.217563 |
|  | 0.25 [a.u.] | 10.87 (7.065 - 27.09) | 17.39 (3.506 - 46.20) | 0.739364 |
| COx_R | 0 [a.u.] | 31.52 (23.52 - 42.39) | 43.48 (6.469 - 57.07) | 0.481251 |
|  | 0.25 [a.u.] | 13.04 (0.5435 - 21.74) | 8.696 (0.0000 - 35.33) | 0.911797 |
